# Specificity and wealth of autobiographical memories in restrictive and mixed anorexic patients

**DOI:** 10.1371/journal.pone.0256959

**Published:** 2021-09-10

**Authors:** Marie-Charlotte Gandolphe, Jean-Louis Nandrino, Marion Hendrickx, Clémence Willem, Olivier Cottencin, Priscille Gérardin, Dewi Guardia, Marie Buttitta, Virginie Zanini, Vincent Dodin

**Affiliations:** 1 Univ. Lille, CNRS, UMR 9193—SCALab—Sciences Cognitives et Sciences Affectives, F-59000, Lille, France; 2 GHICL Service de Psychiatrie, Hôpital Saint-Vincent de Paul, Lille, France; 3 Lille Catholic Hospital, Delegation for Clinical Research and Innovation, Lille, France; 4 Service d’Addictologie, Hôpital Fontan 2, CHRU de Lille, Lille, France; 5 Service de Psychiatrie de l’Enfant et de l’Adolescent, CHU de Rouen, Rouen, France; 6 Clinique Lautréamont, Service de Psychiatrie de l’Adolescent, Loos, France; 7 Univ. Lille, PSITEC Lab EA 4072, Lille, France; University of St Andrews, UNITED KINGDOM

## Abstract

The reduced specificity of positive and negative autobiographical memories observed in anorexic (AN) patients may reflect a global disturbance in their emotional information processing. However, their emotional difficulties may differ according to the subtype of AN, implying possible differences in the manifestation of autobiographical memory impairments. The aims of the study were (1) to confirm the autobiographical memory deficits in AN patients in terms of specificity and wealth of memories, and (2) to compare autobiographical deficits according to the AN subtype: restrictive type (AR) or binge/purging type (AB). Ninety-five non-clinical (NC) individuals and 95 AN patients including 69 AR and 22 AB patients were administered the Williams’ and Scott’s Autobiographical Memory Test. The results confirmed a lack of specificity regardless of emotional valence in the overall AN patient group without any distinction of subtype, which was linked to the number of hospitalizations. When the AN subtype was considered, AR patients demonstrated reduced specificity for negative memories only, suggesting differences in emotional functioning or in the mechanisms underlying reduced specificity between AR and AB patients. Furthermore, the overall AN group demonstrated lower variability and complexity in their memory content than the NC group. However, this difference in the complexity of recalled memories was only found in response to negative cues. When AN subtypes were considered, AR patients showed fewer complex memories than NC individuals. Beyond a reduced specificity, AN patients also depict a poverty in the range of event recall and a difficulty in developing narrative content. The clinical implications of such autobiographical memory deficits need to be further investigated.

## Introduction

Anorexic (AN) patients present specific emotional information processing characterized by rumination and avoidance of experiential and internal states [1–4]. This specific processing is thought to promote the development of dysfunctional schematic representations and to contribute to difficulties in emotion regulation and to symptom maintenance [5–7].

One of the emotional avoidance strategies used by patients with AN is thought to be a tendency to disrupt the recall of specific memories of past events. An individual’s personal memories are known as autobiographical memory (AM) and their role in the construction of the person’s identity and pursuit of goals is central [8–10]. According to the Self Memory System described by Conway & Pleydell-Pearce [8], autobiographical memories are hierarchically organized in the database according to their level of specificity. General memories encompass categoric summaries of repeated events or extended events in time, whereas specific ones are memories of events lasting less than 24 hours that are well located in time and space and are often characterized by their high emotional charge. To avoid the resurgence of emotions associated with past events, a greater tendency to recall general memories at the expense of specific ones has been described in a wide range of mental health disorders such as depression, anxious disorders and addictive disorders as the phenomenon of overgenerality [9,11–13]. Williams was the first to highlight this emotion-regulation aspect of reduced specificity [14] before describing in the CarFAX model that this difficulty in accessing specific memories may also result from ruminative mechanisms and/or from a deficit in executive functioning in some clinical populations of patients [15].

A reduced specificity in response to emotional cue words has already been observed in AN patients [16–19]. Given the emotion regulation impairments that characterize AN patients, this reduced specificity is thought by some to act as a supplementary avoidance strategy used to disrupt the access to emotional content [16,18]. Furthermore, the possible role of ruminative thinking in reduced specificity has also been suggested, as the high accessibility of preoccupying thoughts related to weight and body image may impede the memory specification process by an effect of “capture” [20].

Nevertheless, this impaired recall is not limited to negative memories and has also been observed in response to positive and neutral cues [16,18,19], reflecting the global disturbance of the emotional experience observed in these patients [21]. While reduced specificity may protect patients with AN from the resurgence of emotions in the short term, whatever their valence, this global emotional disruption may lead to a wide range of damaging consequences, as it deprives individuals of the adaptive function of AM. Indeed, a low level of specificity has been related to poorer social support [22], and difficulties in problem-solving [23] and future thinking [24], resulting in impaired decision-making [25]. The link between eating disorders and social problem-solving may also be mediated by memory specificity [20].

The role of reduced specificity in maintaining clinical symptoms in some populations such as patients suffering from depression or addictive behaviors [12,26,27] as well as patients with eating disorders [20] has also been proposed. Furthermore, the clinical outcomes of individuals with reduced specificity might be negatively impacted, as it limits the benefits of therapy [28] and may delay recovery in clinical populations [27]. On the other hand, the duration of illness in patients with AN may reinforce their difficulty in integrating new emotional experiences, resulting in greater difficulty in recalling specific memories. This assumption is supported by the observation of a link between illness duration and specificity [16,18]. However, this link needs to be confirmed as it was not found by all authors [19].

In addition, the onset of AN often occurs during adolescence [29], a key period during which individuals experience life events that will particularly contribute to their identity construction [30]. Thus, the illness and the particular life trajectory resulting from it may have an impact on autobiographical content. They may modify the nature and variety of new experiences, which will then become part of an individual’s life narrative. Furthermore, the particular cognitive and emotional functioning of AN patients characterized by rumination and emotional avoidance may also lead AN patients to recall some memories preferentially at the expense of others that are set aside. Given these considerations, AM deficits in AN patients should also be considered in terms of wealth of memories.

Another concern about AM in AN patients is the observation that deficits occur according to their symptomatology. Studies to date on AM deficits in AN patients were based on a comparison with healthy individuals but did not differentiate between AN subtypes. However, even if restrictive type (AR) and binge/purging type (AB) share some common clinical features, they also have some distinguishable characteristics that go beyond differences in eating-related behaviors (binge/purge versus restriction). For example, AR and AB patients are thought to differ in some cognitive dimensions that may affect AM retrieval such as inhibitory control [31,32] or in their emotion regulation abilities. Indeed, even if the difficulty in emotion regulation is observable in AN patients, differences in the dimensions of emotion regulation according to symptomatology have been evoked [7,33–35]. Consequently, given the difference in emotional information processing between AR and AB patients, the ability to access emotional material such as autobiographical specific memories may also differ. Danner at al. [36] underlined the importance of considering the different eating disorder subtypes instead of considering eating disorder as a whole when assessing emotion regulation strategies. Since reduced specificity may be used as an emotion avoidance strategy, it is relevant to take the different AN subtypes into account.

To our knowledge, no studies have yet addressed this issue. Most studies on AM specificity to date have focused on AR patients [16,18,19] or on bulimia [37] or have not distinguished the subtypes of AN [17].

The objective of the present study was to confirm that specificity is lower in anorexic patients than in non-clinical (NC) individuals both for positive and negative memories. In addition, we aimed to evaluate AM by characterizing the wealth of memories recalled. We hypothesized that patients with AN would recall fewer specific memories whatever the emotional valence (positive or negative), fewer different memories, and that their memories would contain fewer details than those of NC individuals, regardless of emotional valence. Moreover, we examined whether AM in terms of specificity, complexity and differences is linked to clinical variables such as eating disorder symptoms, body mass index (BMI), length of eating disorder, length and number of hospitalizations, and levels of anxiety and depression in AN patients. Finally, we explored whether autobiographical memories are different according to the symptomatology of AN patients and in particular whether there is a difference in the specificity, complexity and percentage of different memories recalled by AR patients, AB patients and NC individuals.

## Material and methods

### Participants and procedure

This study combines data from the pretest phase of the clinical trial OLFANOR and data from a non-clinical group (NC). OLFANOR was a multicenter study conducted in four centers specialized in the treatment of eating disorders in France: the psychiatric and children’s psychiatric department ofSaint Vincent de Paul Hospital in Lille, the addictology department of Calmette Hospital in Lille, the adolescents’ psychiatric department at Lautréamont Clinic in Loos and the adolescents’ and children’s psychiatric department in Rouen Hospital. Each center identified potential participants with an anorexia nervosa diagnosis according to the *Diagnostic and Statistical Manual of Mental Disorders*, fourth edition, text revision (DSM-IV-TR) [38]. A group of 95 hospitalized AN women (mean age 18.34; mean BMI 15.67) composed of 69 AN with AR type (mean age 17.88; mean BMI 15.30) and 22 with AB type (mean age 20.09; mean BMI 16.68) took part in the study. The subtype was not diagnosed for four of them. The study OLFANOR received the approval of the Committee for the Protection of Human Subjects in biomedical research (CPP Nord-Ouest IV; notice number 13/59; IDRCB number: 2013-A01256-39).

A group of 95 non-clinical (NC) women was recruited among the students at a French University (mean age 19.12; mean BMI 20.81). NC participants with a history of eating disorder or a current BMI lower than 17 were excluded from the study. The demographic data of the participants are presented in Table 1.

**Table 1:** Demographic characteristics of NC and AN groups.

	NC group *(n = 95)*	AN group *(n = 95)*	AR group *(n = 69)*	AB group *(n = 22)*	NC vs AN			AR vs AB		
	Means and standard deviations				Value	*P*	Effect size	Value	*p*	Effect size
Age	19.12 (2.63)	18.34 (2.78)	17.88 (2.56)	20.09 (2.89)	*t*(188) = 1.98	*p* = .05	-	*t*(89) = 3.43	*p* = .001	*d* = 0.81
Years of Education					*χ2*(2) = 22.77	*p* < .001	*V* = 0.35	*χ2*(2) = 7.65	*p* < .05	*V* = 0.29
< baccalaureate	15% (*n* = 14)	46% (*n* = 44)	54% (*n* = 37)	23% (*n* = 5)						
= baccalaureate	40% (*n* = 38)	29% (*n* = 27)	26% (*n* = 18)	32% (*n* = 7)						
>baccalaureate	45% (*n* = 43)	25% (*n* = 24)	20% (*n* = 14)	45% (*n* = 10)						
BMI	20.81 (2.34)	15.67 (1.91)	15.30 (1.41)	16.65 (2.37)	*t*(188) = 16.57	*p* < .001	*d* = 2.40	*t*(89) = 3.30	*p* = .001	*d* = 0.69
Length of eating disorder (months)	-	28.01 (26.79)	22.69 (20.42)	45.73 (37.39)	-	-	-	*t*(188) = 3.68	*p* < .001	*d* = 0.76
		Range 3–120	Range 3–120	Range 4–120						
Length of hospitalization (days)	-	65.41 (100.5)	67.18 (88.34)	67.11 (106.91)	-	-	-	*t*(89) = .003	*p* = .99	-
		Range 1–840	Range 14–434	Range 1–840						
Number of hospitalizations (including present one)		1.70 (1.37)	1.65 (1.40)	1.68 (1.32)	-	-	-	*t*(188) = .09	*p* = .93	-
		Range 1–10	Range 1–10	Range 1–6						

All participants were native French speakers. For both groups, participants had to be aged between 13 and 24 years. The exclusion criteria included having a history of substance abuse, psychotic disorder, bipolar disorder or any disturbance that may alter reasoning or understanding abilities such as a neurological disorder, head injury or intellectual deficiency. Participants receiving psychotropic medication were also excluded, except for anti-depressants and anxiolytics if they had been taken for more than 30 days.

A psychiatrist in charge of the study in each center was responsible for selecting the patients who fulfilled the criteria inclusion, whereas control participants were recruited by the research staff. Participation was on a voluntary basis. Prior to their inclusion, the participants had to provide written informed consent and one of their parents or legal representative if they were minors. The research staff presented the study to the participants and administered the tests. The study received the approval of the data protection committee.

### Measures

#### Clinical variables

The participants completed an information sheet on demographic and medical data and the psychiatrist in charge of the study consulted the medical records of the AN group with their consent. The other clinical data were collected with self-administered questionnaires.

The French version of the Eating Disorder Inventory (EDI) [39,40] was used to assess behaviors and psychological characteristics generally related to the presence of eating disorders. This questionnaire consists of a series of 64 items grouped into 8 factors: drive for thinness, bulimia, body dissatisfaction, ineffectiveness, perfectionism, interpersonal distrust, interoceptive awareness and maturity fears. Each answer is rated on a six-point Likert scale ranging from 1 (always) to 6 (never), with a higher score indicating a higher level of eating disorder symptomatology. In our sample, the EDI global score demonstrated an excellent internal validity with a Cronbach’s alpha coefficient of .95. Concerning the various subscales, the internal consistency was good to excellent with a Cronbach’s alpha coefficient of .90 for Drive for thinness, .71 for Bulimia, .89 for Body dissatisfaction, .84 for Ineffectiveness, .77 for Perfectionism, .81 for Interpersonal Distrust, .91 for Interoceptive Awareness, and .75 for Maturity Fears.

Depression and anxiety were assessed using the French version of the Hospital Anxiety and Depression Scale (HADS) [41,42]. This questionnaire consists of 14 multiple-choice questions, half of which explore the level of anxiety and the other half the level of depression. Each item comprises four possible answers scoring from 0 to 3. The score on each subscale (depression and anxiety) is obtained by adding the score on each item. The higher the score, the higher the levels of anxiety and depression. The scores on each subscale indicate a suspicion of a depressive or anxious state when comprised between 8 and 10 and a depressive or anxious state when above 10. In our sample, the internal consistency of each subscale was good with a Cronbach’s alpha coefficient of .83 for the Anxiety subscale, .84 for the Depression subscale and .91 for the global score.

#### Autobiographical memory measures

AM was evaluated with the French version of the Autobiographical Memory Test (AMT) [43] derived from the original test by Williams & Broadbent [44]. This version consists of a list of 10 cue words alternately positive and negative. Each answer was transcribed verbatim in order to be classified either as a general memory or as a specific memory. There was no time limit, and a lack of response (omission) was considered when the participant said he/she had no memory to recall, or when the content of the answer did not correspond to a memory (i.e., "I never feel happy").

The level of specificity, the percentage of different memories and the complexity score were computed from the answers of the participants. The level of specificity corresponds to the proportion of specific memories recalled and was calculated by the formula described by Gandolphe et al. [11]: (Number of specific memories/number of cues—number of omissions) x 100. The proportion of specific memories was also considered regarding the valence of the cues: proportion of specific memories in response to positive cues and in response to negative cues.

Since no instructions on the content of the memories to be recalled were given, the same event may have been mentioned several times by a participant in response to different cue words. To assess the diversity of the recalled memories, the number of different memories, i.e. memories referring to different events, was evaluated and their proportion was calculated with the formula: (number of different memories/number of cues * 100). As the same memory may sometimes be evoked in response to two cues of different emotional valence, the proportion of different memories regarding the emotional valence of the cues could not be examined.

Finally, memories could be evoked with different levels of detail regardless of the level of specificity. The complexity score reflects the amount of information given to describe the recalled past event. Thus, to calculate the complexity score, a basic past event was worth 1 point and each additional detail mentioned represented an additional point.

Example 1 - Specific memory in response to the cue word « successful » with a complexity score of 3: « When I got my driver’s license (basic event– 1 point), I remember it was on a Tuesday (+1 point) and it was a rainy day (+1 point). »

Example 2 –General memory in response to the cue word « happy » with a complexity score of 5: « When my grandparents used to come to see me (basic event– 1 point), it was usually at Christmas (+1 point), they had a long drive to get to my home (+1 point) and we often played board games (+1 point) with my brothers and sisters too (+1 point).

For each participant, the mean complexity score was calculated from all their answers and from answers in response to positive cues and in response to negative cues.

### Statistical analyses

Demographic, clinical and AM characteristics of NC and AN groups were compared. Pearson Chi square tests (χ2) were conducted to compare the level of education, whereas *t*-tests for independent samples were conducted for BMI, age, eating disorder symptomatology (EDI scores), anxiety and depression levels (HADS scores). Concerning the AM variables (AMT scores), the percentage of specific and different memories as well as the complexity score were compared using *t*-tests for independent samples. Concerning specificity and complexity, comparisons were conducted for the percentage of specific memories and the level of complexity among all memories, positive memories and negative memories.

Pearson’s correlation tests were conducted to examine the relationships between AM variables (specificity, complexity, different memories) and clinical variables (eating disorder symptomatology, BMI, length of eating disorder, length of hospitalization, number of hospitalizations, anxiety, depression) in the AN group.

To take the current symptomatology of anorexic patients into account, the AN group was subdivided into two groups (AR and AB) before performing new group comparisons between NC, AR and AB groups. First, a multivariate analysis of variance (MANOVA) using Wilks’ lambda was performed on clinical (eating disorder symptomatology, anxiety and depression levels) and AM variables with the type of disturbance as the between-subject factor. Analysis of variance (ANOVA) was then performed to evaluate the main effect of the type of disturbance on clinical and autobiographical variables before performing post hoc tests. As our sample sizes were unequal, Hochberg’s GT2 pairwise test was used [45].

Finally, *t*-tests for paired samples were conducted to explore the difference in the prevalence of memories recalled according to the emotional valence of the cues, both for specificity and for complexity in each group (NC, AN, AR and AB groups).

For all tests, the level of significance was set at *p* ≤ 0.05. Throughout our statistical analyses and for significant differences, effect sizes were calculated and reported. Cohen’s *d* was used to measure the effect size for significant *t*-test results, with *d* >.20 a small effect size, *d* >.50 a medium effect size and *d* >.80 a large effect size [46]. For significant Chi-square results, Cramer’s V was calculated, with an effect size considered as small when.07 < V < .21, medium when.21 < V < .35 and large when V >.35 in the case of *df* = 2 [47]. Partial eta-squared (partial η2) was used to estimate the effect size of significant ANOVA results. The effect size was small when η2 >.01, medium when η2 >.06 and large when η2 >.14 [47]. All analyses were conducted using SPSS software version 24 for Windows. For all questionnaires, the missing data were replaced by the average of the non-empty items (Siddiqui & al. 2015). Before, the rate of missing values for each questionnaire was calculated for each patient and considered as reasonable (<30%). Data are available in S1 Dataset.

## Results

### Anorexic and non-clinical group comparisons

Group comparisons between NC and AN groups are presented in Table 3. The paired-sample t-test revealed that NC individuals differed significantly from AN patients for all clinical and autobiographical variables. NC individuals presented less eating disorder symptomatology measured by the EDI than AN patients, except for bulimia. Furthermore, anxiety and depression levels measured with the HADS were lower for NC individuals than for AN patients.

Concerning autobiographical variables measured with the AMT, NC individuals recalled more specific memories, regardless of their emotional valence. The effect size was large when all memories and only negative memories were considered, while it was small to medium with positive memories. Furthermore, NC individuals recalled more different memories than AN patients, with a medium to large effect size. The complexity score was also higher for the NC group than for the AN group. This result was also found for both positive and negative cues, with a small to medium effect size.

### Correlations

Pearson’s correlation coefficients conducted to explore the relationships between autobiographical and clinical variables are shown in Table 2. In the AN group, regardless of the subtype considered, the results showed that the level of specificity was significantly and negatively related to the number of hospitalizations, suggesting that the more AN patients experienced episodes of hospitalization, the less they recalled specific memories. This result was also observed for AR patients. Furthermore, in the AR group, the level of specificity was also linked to the BMI, with a higher BMI associated with more specific memories recalled.

**Table 2:** Correlations between clinical and autobiographical memory variables.

	NC Group			AN Group			AR Group			AB Group		
	Level of specificity	Different memories	Complexity	Level of specificity	Different memories	Complexity	Level of specificity	Different memories	Complexity	Level of specificity	Different memories	Complexity
EDI total	.04	-.10	-.25*	.05	-.24*	.06	.24*	-.26*	.06	-.15	-.05	.06
Drive for thinness	.13	-.02	-.12	.06	-.19	-.03	.21	-.15	-.08	-.14	-.22	.22
Bulimia	-.11	-.14	-.04	-.12	-.08	-.03	.12	-.15	-.05	-.04	.05	.27
Body dissatisf.	.07	-.01	-.13	.05	-.29**	.01	.16	-.28*	.02	-.05	-.29	-.05
Ineffectiveness	-.12	-.10	-.19	.03	-.26**	.07	.18	-.28*	.10	-.16	-.15	-.01
Perfectionism	.10	.05	.04	.05	-.07	.06	.22	-.08	.09	-.18	.08	-.15
Interpers. Distress	-.04	-.17	-.30**	.06	-.12	-.01	.15	-.20	-.03	-.04	.18	-.06
Intero. awareness	-.03	-.12	-.22*	.12	-.17	.10	.23	-.20	.08	.07	-.01	.10
Maturity Fears	.03	-.06	-.18	-.04	-.03	.12	.05	-.07	.13	-.25	.20	-.07
HADS total	-.06	-.11	-.12	.12	-.31**	.06	.21	-.31*	.03	.18	-.24	.07
Anxiety	-.04	-.08	-.13	.14	-.26*	.06	.20	-.29*	.01	.30	-.01	.10
Depression	-.06	-.11	-.06	.06	-.32**	.06	.19	-.29*	.05	.01	-.41	.02
BMI	.10	-.02	-.09	.12	-.07	-.03	.29*	-.10	-.03	.03	.06	.11
Length of eat. dis.	-	-	-	.04	-.02	.05	-.01	.03	.12	.18	-.23	-.28
Length of hospit.	-	-	-	-.03	.04	.09	-.03	.005	.08	-.09	.16	.10
Number of hospit.	-	-	-	-.22*	.02	.06	-.32*	-.01	.16	.06	.04	-.34
Different memories	.12	1	.31*	.15	1	.29**	.03	1	.30*	.38	1	.34
Complexity	.23*	.31*	1	.004	.29**	1	-.08	.30*	1	.27	.34	1

*p < .05;

**p < .01.

Furthermore, there were significant negative correlations between the percentage of different memories and certain EDI scores, as well as HADS scores in the AN group and in the AR group. Consequently, the less patients with AN and especially AR tended to recall different memories, the more they demonstrated eating disorder symptoms, especially body dissatisfaction and a feeling of ineffectiveness, and the higher were their levels of anxiety and depression.

Finally, there were significant negative correlations between complexity and certain EDI scores in the control group. The less complex their memories were, the more they felt interpersonal distress and a lack of interoceptive awareness.

Regarding the link between AM variables, there was no link between specificity and the proportion of different memories, regardless of the group considered. A higher level of specificity was linked to more complexity in the NC group. A higher level of complexity was linked to a higher level of specificity in the NC group and to a greater proportion of different memories in the overall AN group and in the AR subgroup.

### Effect of current type of eating disorder on clinical and autobiographical variables

Using Wilks’s lambda, there was a significant effect of the type of disturbance on eating disorder symptomatology, anxiety, depression and AM measures, Λ = .21, F_(26,330)_ = 14.93, *p* < .001, η2 = .54. Using a separate univariate ANOVA, a main effect of the type of disturbance on eating disorder symptomatology measured by the EDI was found, whatever the subdimension: EDI total (F_(2,183)_ = 83.45, p < .001, η2 = .48), drive for thinness (F_(2,183)_ = 49, p < .001, η2 = .35), bulimia (F_(2,183)_ = 62.78, p < .001, η2 = .41), body dissatisfaction (F_(2,183)_ = 38.92, p < .001, η2 = .30), ineffectiveness (F_(2,183)_ = 52.38, p < .001, η2 = .36), perfectionism (F_(2,183)_ = 25.80, p < .001, η2 = .22), interpersonal distrust (F_(2,183)_ = 22.04, p < .001, η2 = 19), interoceptive awareness (F_(2,183)_ = 70.46, p < .001, η2 = .43), maturity fears (F_(2,183)_ = 17.57, p < .001, η2 = 16). ANOVA results also showed a main effect of the type of disturbance on anxiety and depression: HADS total (F_(2,183)_ = 49.18, p < .001, η2 = .35), HADS anxiety (F_(2,183)_ = 36.46, p < .001, η2 = .28), HADS depression (F_(2,183)_ = 45.72, p < .001, η2 = .33).

Hochberg’s GT2 post-hoc test results are presented in Table 3. Concerning eating disorder symptomatology, NC individuals demonstrated fewer eating disorder symptoms than the AR and AB groups. The two groups of anorexic patients did not differ in terms of body dissatisfaction, ineffectiveness, interpersonal distrust and maturity fears. On the other hand, AB patients demonstrated a higher EDI total score, with more bulimia, perfectionism and interoceptive awareness deficits than AR patients. Only the dimension drive for thinness was significantly higher in AR patients than in AB patients.

**Table 3:** Descriptive statistics and group comparisons (*t* test or Hochberg’s GT2 pairwise test) between NC and anorexic groups for clinical and autobiographical memory variables.

	NC group *(n = 95)*	AN group *(n = 95)*	AR group *(n = 69)*	AB group *(n = 22)*	NC vs AN			NC vs AR	NC vs AB	AR vs AB
	Means and standard deviations				*t* Value	*p*	Cohen’s *d*	Hochberg’s GT2 pairwise test		
EDI total	34.95 (17.82)	79.16 (33.28)	73.22 (32.79)	100.18 (24.44)	11.41	p < .001**	1.65	p < .001**	p < .001**	p < .001**
Drive for thinness	3.61 (4.52)	11.25 (6.74)	10.32 (6.99)	4.45 (4.62)	9.18	p < .001**	1.33	p < .001**	p < .001**	p < .01*
Bulimia	3.25 (2.03)	2.35 (4.19)	0.58 (1.55)	7.09 (5.17)	1.89	p = .06	-	p < .001**	p < .001**	p < .001**
Body dissatisfaction	6.93 (4.95)	15.16 (8.07)	14.42 (8.21)	17.68 (6.99)	8.47	p < .001**	1.23	p < .001**	p < .001**	p = .13
Ineffectiveness	4.80 (3.48)	12.13 (6.37)	11.43 (6.62)	14.27 (4.71)	9.83	p < .001**	1.43	p < .001**	p < .001**	p = .06
Perfectionism	4.44 (3.83)	8.05 (4.20)	7.49 (4.06)	10.41 (3.95)	6.19	p < .001**	0.90	p < .001**	p < .001**	p < .01*
Interpersonal Distrust	3.38 (3.64)	7.11 (4.40)	6.97 (4.32)	8.14 (4.72)	6.36	p < .001**	0.92	p < .001**	p < .001**	p = .56
Interoceptive Awar.	3.31 (3.83)	13.71 (4.42)	12.78 (8.34)	17.68 (7.76)	10.95	p < .001**	2.51	p < .001**	p < .001**	p < .01*
Maturity Fears	5.23 (4)	9.41 (5.82)	9.22 (5.95)	10.45 (5.59)	5.76	p < .001**	0.84	p < .001**	p < .001**	p = .67
HADS	11.97(4.51)	20.59 (7.90)	19.72 (8.32)	23.95 (5.35)	9.24	p < .001**	1.34	p < .001**	p < .001**	p = .19
HADS anxiety	8.61 (2.90)	13.12 (4.98)	12.62 (5.27)	15.41 (3.16)	7.62	p < .001**	1.11	p < .001**	p < .001**	p = .14
HADS depression	3.36 (2.44)	7.47 (3.56)	7.10 (3.59)	8.55 (3)	9.30	p < .001**	1.35	p < .001**	p < .001**	p = .14
AMT										
Specificity										
Total memories	78.09 (16.14)	62.61 (21.23)	64.78 (20.16)	56.27 (24.25)	6.66	p < .001**	0.82	p < .001**	p < .001**	p = .18
Positive memories	75.95 (18.26)	65.23 (26.33)	67.41 (25.51)	58.86 (29.60)	3.26	p = .001**	0.47	p = .054	p < .01**	p = .33
Negative memories	81.35 (19.26)	60.10 (24.46)	62.60 (23.84)	52.72 (25.30)	6.65	p < .001**	0.96	p < .001**	p < .001**	p = .18
Different memories	96 (5.72)	90.84 (11.73)	91.30 (11.87)	89.09 (12.31)	3.85	p < .01**	0.56	p < .01**	p < .01**	p = .70
Complexity										
Total memories	4.05 (1.39)	3.49 (1.16)	3.51 (1.20)	3.58 (1.07)	2.94	p < .001**	0.44	p < .05*	p = .35	p = .99
Positive memories	3.92 (1.42)	3.47 (1.25)	3.47 (1.26)	3.64 (1.30)	2.30	p = .02*	0.47	-	-	-
Negative memories	4.17 (1.53)	3.52 (1.26)	3.55 (1.33)	3.53 (1.01)	3.19	p = .002**	0.46	p = .02*	p = .16	p = 1

*p < .05;

**p < .01.

Regarding autobiographical variables, a main effect of the type of disturbance was found as determined by a one-way ANOVA for specificity (F_(2,183)_ = 17.29, p < .001, η2 = .16), frequency of different memories (F_(2,183)_ = 7.82, p = .001, η2 = .08) and complexity (F_(2,183)_ = 3.75, p = .02, η2 = .04).

When positive and negative cues were considered separately, we found a main effect of the type of disturbance on specificity for both positive (F_(2,183)_ = 6.27, p = .002, η2 = .06) and negative memories (F_(2,183)_ = 23.54, p < .001, η2 = .20), and on complexity for negative memories (F_(2,183)_ = 4.53, p = .01, η2 = .05) but not for positive ones (F_(2,183)_ = 2.29, p = .10). Consequently, post-hoc tests were not conducted concerning the complexity score for positive cues.

Hochberg’s GT2 post-hoc tests revealed that NC individuals showed more specific and more different memories than AR and AB patients, while they differed only from AR patients for complexity when the emotional valence of the cues was not considered.

However, NC individuals recalled more specific memories in response to positive and negative cues than AB patients and AR patients for negative cues only. NC individuals also recalled more complex memories than AR patients but not AB patients in response to negative cues.

Finally, the two groups of anorexic patients did not differ in terms of specificity, different memories and complexity, regardless of the emotional valence considered.

Concerning the difference in the prevalence of memories recalled according to the emotional valence of the cues in each group, only NC individuals demonstrated a higher frequency of specific memories recalled in response to positive cue words than in response to negative ones (*t*_(94)_ = 2.33, *p* = .02, *d* = .24). There was no difference in the prevalence of specific memories recalled according to the valence of the cue in the other groups. There was no difference either in the level of complexity when memories were recalled in response to positive or to negative cues in any group.

Post-hoc tests revealed that NC individuals showed lower levels of anxiety and depression than the AR and AB groups. However, the two groups of anorexic patients did not differ in terms of anxiety or depression levels. Concerning AM, NC individuals recalled more specific memories than patients, with no difference between the AR and AB groups. However, when specificity was compared by taking into account the valence of the memories, the results were the same for negative memories, whereas AR patients did not differ either from NC individuals or from AB patients concerning the percentage of specific memories recalled with positive cues. In contrast, AB patients recalled fewer specific memories for positive cues than NC individuals.

NC individuals recalled more different memories than the two groups of anorexic patients, which did not differ from each other. The complexity score was significantly higher for NC individuals than for AR patients, whereas the score of AB patients do not differ from those of the NC and AR groups.

## Discussion

This study examined AM recall in AN patients in terms of specificity and wealth of memories. The results confirm that when AN subtypes are not considered, AN patients display a reduced level of specificity that is not related to depressive symptoms, which is in line with previous studies [16,18,19]. This lack of specificity in AN patients was observed whatever the valence of the memories considered and reflects a global emotional disturbance in these patients [16]. However, regarding the actual type of anorexic episode, specificity was lower in both AR and AB patients for negative memories, whereas AR patients seem not to demonstrate less specificity than NC individuals for positive memories, even if this finding should be interpreted with caution due the value of p, which is close to .05. Yet in most previous studies AR patients found it difficult to recall specific memories with both positive and negative cues [16,18,19], even though one study demonstrated no difference between AN patients and an NC sample in the recall of positive content in an autobiographical task [48]. This divergence may be partly due to the different clinical characteristics of the samples such as BMI, illness duration [48] and involvement in a psychotherapy, which is known to influence the specificity of recall [49].

In our sample, the difficulty in accessing specific memories in AR patients is mainly explained by a difficulty in accessing negative memories. This result is in line with the affect regulation hypothesis initially developed by Williams [14], i.e. recalling events at a general level is an attempt to avoid the resurgence of negative emotions that may occur with the reactivation of painful events from the past. This may explain why the lack of specificity mainly concerns negative memories, especially in populations of patients who have difficulty in regulating their emotions. Furthermore, in AR patients, a higher BMI is associated with more specific memories (and inversely). This result raises the question of whether having a low weight and engaging in restrictive behaviors leading to a low weight may impact AM specific recall. Brockmeyer et al. found that food restriction and weight loss were associated with lower negative recall in an autobiographical task [48]. They argued that these distorted eating behaviors attenuated the recall of painful emotional experiences. Consequently, they may be considered as emotion regulation strategies used by AN patients, in parallel to reduced specificity.

In addition, the link between BMI and the level of specific recall questions the impact of starvation on cognitive functioning and consequently on AM. Although the role of cognitive impairment in AM recall has rarely been debated in AN patients, it might be another factor contributing to reduced specificity that should be explored. This link between BMI and specificity was not found in AB patients, in whom the recall of specific memories was impaired for both positive and negative memories. Consequently, variables potentially related to BMI such as weight, eating behaviors and cognitive functioning may be less involved in the lack of specificity for AB than for AR patients. The lack of specificity observed regardless of emotional valence may reflect a more global emotional disturbance in AB patients. It may be due to a lesser ability to mobilize resources to deal with emotions triggered by the recall of both positive and negative past events. This is consistent with the observation of difficulties in using adaptive emotion regulation strategies, such as cognitive reappraisal in AB patients [36]. This supports the hypothesis of different emotional functioning in the two subtypes of AN patients, which might account differently for the lack of specificity. However, further studies including the assessment of emotion regulation abilities are needed to explore how emotional deficits account for AM recall deficits in AR and AB patients. Furthermore, the difficulty in accessing emotional content in AB patients whatever the valence of the memories might also reflect a shift in reduced specificity towards an automatic mode of response. This might result from the repeated avoidance of emotion, independently from the valence of the autobiographical content [50]. The longer duration of illness in AB patients than in AR patients would support this assumption.

While reduced specificity in patients with AN is often considered as a way to avoid emotions, there is no empirical evidence to confirm this assumption. Other mechanisms might be involved, as suggested by the CaRFAX model [15]. The involvement of ruminative functioning in the difficulty to access specific autobiographical content in individuals with eating disorder has also been suggested [20]. In AN patients, ruminative thinking might be associated with dysfunctional schematic representations of self and one’s environment especially in relation to food control, weight loss and shape, on which patients remain focused [5]. Given the salience of these thoughts, patients might be prone to them whenever they engage in an autobiographical search process, thereby impeding the access to specific memories. In this vein, Bomba et al. [18] found that AN patients experienced greater difficulty in recalling specific memories in response to cues related to eating symptoms and behaviors. However, while the lack of empirical studies makes it difficult to draw conclusions, the involvement of abstract ruminative thoughts in reduced specificity needs further investigation.

Beyond this lack of specificity, our results show that AM impairments in AN patients are also reflected by a lower complexity score and by a lower proportion of different memories recalled than in NC individuals. The difficulty to recall different memories was observed whatever the subtype of AN, whereas a lower complexity was mainly observed in AR patients as AB patients did not differ from AR or NC individuals. Furthermore, the lack of complexity was only found for memories recalled in response to negative cues. This difficulty to recall negative complex memories in AR patients seems consistent with the specificity results showing reduced specificity in response to negative cues in these patients. These deficits seem to reflect distancing from the negative emotions associated with recalling painful events in AR patients. However, they seem to be able to recall details from positive events, whose emotional charge might be easier to deal with.

The absence of significant differences between AB patients and control individuals in terms of complexity may be surprising, especially given the similar mean values for this variable in the AR and AB groups. This value was even lower in the AB group than in the AR group regarding the level of complexity for negative cues. However, the small size of the AB sample or the presence of outliers in the AN group may have influenced the results, which underlines the necessity to replicate the study with a larger sample of the AB subgroup. If the results are confirmed, they may suggest that disruption of emotional autobiographical content as an emotion regulation strategy may be used at a different level or in a different way in AN patients according to their subtype. This would support the theory of different emotional functioning.

Regardless of the group of participants considered, the level of specificity was not related to the number of different memories, suggesting that they may be considered as independent constructs. Indeed, individuals may recall specific memories with a poor diversity of events recalled in response to the various cue words. On the other hand, the level of specificity was linked to the complexity score in the control group, suggesting that individuals from the general population who tend to recall more specific memories are also able to recall more details when reporting past events. This link was not found in AN patients, as it is possible to recall specific memories with a poor description of these events.

Not only is AM truncated at a general level in AN patients; their memories are also less varied and are underdeveloped. Surprisingly, deficits in AM recall do not depend on the duration of the eating disorder but rather on the number of hospitalizations, which seems to affect the level of specificity. Therefore, having an eating disorder is not in itself what impairs the recall of specific memories, but rather how the illness was experienced and whether it hinders the patient’s daily life and pursuit of goals, particularly repeated hospitalizations. In addition, beyond experiences of life such as hospitalizations that might affect autobiographical recall, the role of rumination and emotional avoidance should also be considered in the lack of rich memories. The latter may result from repeated thinking about concerns that may or may not be linked to their illness, especially since the more AN patients were anxious and depressed, the less they recalled different memories. Furthermore, avoiding the recall of some events associated with painful emotions could limit the autobiographical content of memories.

The clinical implications of this lack of autobiographical content are intriguing and have never been studied in AN patients. In other clinical populations, such as depressive patients, reduced specificity is associated with a more negative clinical outcome and a longer delay to remission [27]. Furthermore, a lack of specificity may itself increase the risk of depression by locking the individual into undifferentiated and negative self-schemes that enhance ruminative negative thinking [15]. In other addictive behaviors such as substance abuse disorders, poor AM content may impact personal adjustment negatively, resulting in a greater risk of relapse. This could be due to the individual’s inability to learn from past experiences, but also to a difficulty in accessing personal achievements that would improve self-esteem and motivation [12,51]. The consequences of AM deficits on the construction of identity and on the adaptability of AN patients need further investigation, as well as their involvement in treating their eating disorder and ruminative functioning.

Finally, our results confirm that specificity is reduced in AN patients, whatever the subtype. They also underline some impairments in other dimensions of autobiographical content, especially a poverty in the range of event recall and a difficulty in developing narrative content. These findings should be considered in light of some limitations, especially in the constitution of our groups. First, our AN group had a lower level of education than our control group. However, being hospitalized may hinder the pursuit of education in AN patients, whose mean age (18 years old) is the typical age when they take the French baccalaureate examination. Although this impact on educational level may no longer exist in an older group of AN patients, this issue still needs to be explored. Second, the two subtypes of AN patients differed in their clinical characteristics, especially their age and illness duration. Although these differences are difficult to control for because the groups were constituted from diagnostic categories and were not randomly assigned [52], this raises the question of the impact of these variables on AM recall. Indeed, illness duration might impact the nature and variety of experienced life events, as well as the emotional and cognitive functioning of the individual whose implication in AM recall has already been underlined. Age difference may also impact AM recall as episodic memory abilities and autobiographical subjective experience evolve across adulthood, even though autobiographical content itself has been shown to be independent from age [53,54]. However, in the present study, the effect of age on AM recall should be put into perspective since the participants were in the same life period, with an average age between 18 and 20 years old. Finally, the effect of therapy received by hospitalized patients on AM recall has not been assessed, so it would be interesting to explore this potential variable.

Despite these limitations, the rehabilitation of AM should be a therapeutic target in AN patients, given its likely deleterious effects on their adaptability and remission. Indeed, recalling more general, less developed and less varied memories is likely to lock AN patients into dysfunctional and often negative representations of themselves and their environment, which in turn contribute to maintaining cognitive bias. Autobiographical deficits may also contribute to poor self-consideration under the lens of the disease, which may negatively affect self-esteem and hinder change. Focusing on a restricted range of general past events prevents AN patients from enjoying the adaptive function of AM in everyday life, leading them to adopt the same dysfunctional behaviors and strategies in an inflexible way (such as dysfunctional eating behaviors or emotional avoidance). Furthermore, autobiographical deficits have already observed in these patients during adolescence [18], a key period marked by important events and life choices, and during which personal goals are often defined. Identity development may thus be particularly affected by the lack of specificity and wealth of AM in adolescent patients. This underlines the major clinical issues related to the development of therapeutic approaches focusing on AM in AN patients. In other populations of patients in which AM recall deficits were observed, such as depressive patients, memory specificity training has been shown to positively impact the specificity of AM recall as well as emotional and cognitive factors involved in both depression and AM deficits, such as rumination or emotional avoidance [55–57]. Programs such as the MEST (memory specificity training) or the MEMFlex have shown significant results in depressive inpatients [55,57], in adolescents with depressive symptoms and aversive experiences [56] and in veterans with post-traumatic stress symptoms [58]. These results open up new therapeutical perspectives in the treatment of AN patients [57].

## Supporting information

S1 Dataset Demographical and clinical variables Dataset for anorexia and control groups. (XLSX)
